# Health education provided by nurses to children and young people: parents’ assessment

**DOI:** 10.1186/s12912-023-01447-x

**Published:** 2023-08-25

**Authors:** Anabela Fonseca Pereira, Joaquim José Jacinto Escola, Carlos Manuel Torres Almeida, Vítor Manuel Costa Pereira Rodrigues

**Affiliations:** 1 Higher School of Health, Portuguese Red Cross Alto Tâmega, Chaves, Portugal; 2 Institute of Philosophy of the University of Porto, School of Human and Social Sciences, University of Trás-os-Montes e Alto Douro, Vila Real, Portugal; 3 Research Center in Sports Sciences, Health Sciences and Human Development, CIDESD Clinical Academic Center of Trás-os-Montes e Alto Douro Doctor Nuno Grande-CACTMAD Vila Real, School of Health, University of Trás-os-Montes e Alto Douro, Vila Real, Portugal; 4 Research Center in Sports Sciences, Health Sciences and Human Development, CIDESD, Clinical Academic Center of Trás-os-Montes e Alto Douro Doctor Nuno Grande-CACTMAD Vila Real, School of Health, University of Trás-os-Montes e Alto Douro, Vila Real, Portugal

**Keywords:** Child, Health education, Health promotion, Nursing

## Abstract

**Background:**

Healthy literacy is a determinant key children/teenager’s health and health outcomes. The aim of this study to identify the parents’ assessment about Health Education practice to children and teenagers.

**Methods:**

We opted for a descriptive, quantitative and cross-sectional research, with a non-probabilistic convenience sample. The inclusion criteria were: being a parent who uses attending children health appointments in primary health care; being a parent who has a child hospitalized and is accompanying him/her in the pediatric hospital inpatient ward. A questionnaire survey was built with three sections: sample characterization, Health Education practices performed by nurses (5 questions) and a scale that measured Health Education Assessment Scale (HEAS), which contained 48 items and was validated. It was applied from September to December 2018.

**Results:**

The survey was filled in by 113 parents. The results showed that 100% (*n* = 113) of the parents feel comfortable to talk with nurses about children/teenagers health; 79.6% (*n* = 90) consider that nurses have time availability for the doubts clarification; 61.9% (*n* = 70) point out that nurses identify child/teenager needs; Healthy eating” (60.2%; *n* = 68), the “National Vaccination Plan” (53.1%; *n* = 60) and “Harmful behaviors prevention” (46.9%; *n* = 53) are the most important topics; 56.6% (*n* = 64) of the parents, when in doubt, turn first to the pediatrician, and 66.4% (*n* = 75) considered that this practice was equal important, compared with other nursing interventions.

**Discussion:**

This study shows that Health Education provided by nurses is based on the need’s identification, with a perspective of involvement and participation, promoting health and conscious changes which reinforces the nurses’ position as health educators.

## Background

According to World Health Organization (WHO), Health Promotion (HP) directed to teenagers has great potential to promote the population health, leading to the promotion of healthy behaviors, which makes it an effective way for children, teenagers, and families to exercise a greater control over their health and contribute to its improvement [1]. WHO also promotes an integrated approach to managing childhood illness that considers all aspects of a child’s health, and a continuum of care throughout the early years to safeguard their developmental outcomes, including the reduction of risk factors for diseases that can arise later in life [2].

Thus, Health Education (HE) can be understood as a process in which individuals or groups learn to promote, maintain, or restore health [3], assuming itself as a practice that contains a set of consciously constructed opportunities, in which some form of communication is used, and which aims to promote health literacy, increase knowledge and attitudes that lead to individual and community health [4].

In this sense, it is important to consider that parents and caregivers play a key role in promoting their children’s health and well-being [5], and, therefore, nurses help them with the developing of their parenting skills and their ability to perform effective interventions on their children [6]. In turn, the literature also points the consensus that more health literacy, more reduction of erratic behaviors and, consequently, better health gains. On the other hand, this literacy helps parents and caregivers to use health services more effectively [5].

On this basis, the interventions’ effectiveness depends on their suitability to the target audience [3], and therefore, the literature points the nurse, as the health professional with a broad role that identifies problems, interrupts negative development trajectories, promotes healthy behaviors and lifestyles, and improves social inclusion using a strengthening community participation [7]. In this context, considering that nurses are the health professionals more involved in HE interventions, it’s important to point out the benefit of the individual/community along with their interventions [8] and the research that is also needed to identify or develop effective nursing practices to eliminate gaps and disparities in health care [9].

The HE practice usefulness is highlighted in several studies [1, 3, 5, 6] and scientific literature, and nurses’ role in leading the HE strategies adoption is also highlighted, thus making a decisive contribution in increasing the population’s health literacy and promoting the ability of informed health decisions [7]. Although, according to a review, *literature the evidence-based practice*, from the 1990s, there are serious concerns that needs to be addressed, such as the practice wisdom and the nursing practice complexity, so it is important the reflection of nursing practices [10].

In parallel, parents’ perspectives are also seen as an important component of internationally evidence-based practices which ensure the health services quality [11], so it is important that nurses demonstrate develop quality care that meets safety standards with satisfactory patient results [12].

Thus, assuming that to build a better future, society has to invest in children [2]. The nurses are the foundation of all health systems in the world, and, therefore, in the best position to influence the individuals and community health well-being [13], and parents, as partners in care, can assess the relational skills and health professional’s knowledge [12].

### Health promotion in Portugal

In the last decade, the Portuguese health status has improved considerably, although health inequalities are linked to a series of determinants of health (stress, people’s conditions, physical environment) and behavioral risk factors (tobacco, alcohol, diet and physical inactivity) [14]. For example, children up to 6 years of age, there are programs with intersectoral responsibility and interdisciplinary approaches performed by nurses [14]. There are also programs with the objective of promoting health decision making, self-confidence and the mental well-being of vulnerable pre-adolescents, also performed by nurses [14]. In this scenario, the HE practice stands out as a valuable tool, as nurses play an important role in empowering the individuals in their health promotion, thus, both the role of nurses as educators in the health promotion process and their technical and human capacity to meet the individuals and family’s needs are recognized [14].

## Methods

### Study design and study population

The present study has the general objective to identify the HE practice evaluation carried out by nurses to children, teenagers and parents. As a specific objective, we intended to identify the assessment made by parents about the HE practice, provided by nurses, to children, teenagers and parents. In this sense the aim of this study is clarify the contribution of HE practice carried out by nurses in children, teenagers and parent’s health.

This is a quantitative and cross-sectional research, developed in two different areas: differentiated health care and primary health care, from September to December 2018. For this purpose, we used pediatric services in a total of 4 hospitals and 29 health centers units in Northern Portugal. The inclusion criteria were: being a parent who uses attending children health appointments in primary health care (up to 18 years), or being a parent who has a child hospitalized and is accompanying him/her in the pediatric hospital inpatient ward (up to 18 years).

It should be noted that our first objective was to understand how parents evaluated HE practice in a global view. Since HE is carried out at the various levels of care (primary, differentiated) and parents can even have experience of the various aspects, we understand that we should have a global assessment of the importance attributed to HE regardless of the place where is done, trying to do a separate analysis afterwards. In this sence we had a non-probabilistic sampling of convenience, consisted of 113 parents who gave their consent.

Based on the objectives, and having as the main starting point, the conceptual context, the target population and the type of study, the hypotheses of this research are: the assessment that parents/family attribute to the HE practice provided by nurses to children and teenagers varies according to their academic/professional qualifications; the assessment that parents/family attribute to the HE practice provided by nurses to children and teenagers varies according to their age; the evaluation that parents/family attribute to the HE practice provided by nurses to children and teenagers varies according to their academic/professional qualifications; the evaluation that parents/family attribute to the HE practice provided by nurses to children and teenagers varies according to their age.

### Data collection

To build the questionnaire survey an extensive literature review was carried out through bibliographic research related, to the theoretical and social context of the phenomenon under study.

An analysis of knowledge in nursing was also carried out based on scientific works archived in institutional repositories with free access, looking for scientific productions with a central focus on HE practice carried out by nurses to the children, teenagers, and parents in Portugal.

The databases used were: Online Knowledge Library (B-On); EBSCOhost Online Research Databases (EBSCO); Psychology and Social Science Journals on the Web (PSYCLINE); Medical Literature Analysis and Retrieval System Online (MEDLINE); Scientific Electonic Library Online (SciELO); ELSEVIER; PubMed Central (PMC); Portuguese Open Access Scientific Repository (RCAAP). The reading and analysis of relevant strategic documents was also carried out, such as: documents from official international (WHO, ICN, UNESCO, UNICEF) and national organizations.

The choice of this plurality of bibliographic references resulted from the search of the most representative for the context of this study, due to the scarce information in HE practice for the children, teenagers and parents disclosed in Portugal, and the lack of a research in Portugal with the same object of study. Finally, in order to prepare the data collection instrument, it was considered an important added value, to perform interviews to experts in the field of HE practice to hearing reflections and opinions, and parents to get their evaluation. In this sense, semi-structured interviews were conducted (using a grid of open and standardized questions) with 10 nurses and 20 parents, in order to obtain aspects that the researchers would not have thought of and complete the bibliographic review. We opted for a non-probabilistic convenience sample in order to have more access to people, and these answers were subjected to a content analysis, and also an analysis by five experts (two PhD Professors in Nursing, two researchers with extensive experience in building scales, and two Nursing professionals with wide experience in HE). Once the variables that allow responding to the study’s problem were defined, the questionnaire elaboration was completed.

The questionnaire was constituted by 3 sections: sample sociodemographic characterization (3 questions); HE practices provided by nurses’ characterization (5 questions); HE practice provided by nurses’ evaluation (1 question and 1 scale) (Table 1). Since no questionnaire was found that responded to the problem of this research, a scale which measures HE provided by nurses - Health Education Assessment Scale (HEAS) which contained 48 items, was built and validated [15].

**Table 1:** Questionnaire used with the participants

Sociodemographic characterization	1. Age 2. Gender 3. Academic/professional qualifications
HE practices provided by nurses’ characterization	1. Do you feel free to talk with nurses about you child health? (Yes/No) 2. Do you feel that nurses have time availability to clarify doubts about your child health? (Yes/No) 3. Does it seem to you to be a prepared approach? (Prepared according to guidelines/Preparation according to identified needs/Without preparation and using improvisation/Other) 4. What themes do they address most? (Healthy eating/Accidents prevention/Nacional Vaccination Plan/Child development/Bullying/Personal hygiene/Sleep routines/Oral health/First aid/Harmful behaviors prevention/sexual education/Other) 5. who do you ask first, when you have child’s health doubts? (Family’s doctor/Pediatrician/Nurse/Other)
HE practice provided by nurses’ evaluation	1. What is the importance of HE practice provided by nurses? (Not important/Less importance/Equal importance/Greater importance) 2. HEAS (Health Education Assessment Scale)

### Ethical statement

In data collection, in primary health care, the questionnaire was completed by parents in the consultation room, at the end of the children health appointments. At the pediatric hospital, the questionnaire was distributed by nurses and completed by parents in the ward.

In order to follow the ethical requirements, the research was validated by the institutions ethics committees involved, and the questionnaires were authorized by the directors (authorization n. 124/2018; n. 256/2018 and n. 00316/2018). When distributing the questionnaire to the participants, the informed consent document was provided to them, and it was explained and ensured the guarantee of their privacy, anonymity and confidentiality of the collected data.

### Statistical analyses

The Statistical Package for the Social Sciences (SPSS) Version 22.0 was used for statistical analysis. A descriptive analysis was performed using central tendency measures, dispersion and frequency distribution. For inferential analysis, and, taking into account that the sample did not reveal normal distribution (Kolmogorov-Smirnov test), non-parametric tests were used (chi-square (χ) test [16] by Monte Carlo simulation, as well as Kruskal-Wallis test), assuming a significance level of 0.05 (*p* < 0.05).

## Results

### Professional and sociodemographic sharacterization

The sample is mainly composed by women (94.7%; *n*=107) and only 5.3% (*n*=6) by men. The age range that enclosed the highest number of respondents (49.6%) was 30-40 years. The mode was the age interval of 30-40 years. Regarding academic/professional qualifications, 54 of the respondents (47.8%) had a "Degree", 12 (10.6%) had a "Master's Degree", 13 (11.5%) had a "3rd Cycle (9th year)", 20 (17.7%) had a "12th Year" and 14 (12.4%) had a "Professional Course" (Table 2).

**Table 2:** Sociodemographic and professional characterization of the sample. Northern Portugal, 2018, (*N*=133)

Professional and sociodemographic characterization		n*	%
Gender	Male	6	5,3
	Female	107	94,7
Age	20-30 years	20	17,7
	30-40 years	56	49,6
	40-50 years	36	31,9
	50-60 years	1	0,9
	>60 years	-	-
Academic/professional qualifications	4th grade	-	-
	6th grade	-	-
	9th grade	13	11,5
	12th grade	20	17,7
	Professional course	14	12,4
	Graduation	54	47,8
	Master	12	10,6
	Doctorate	-	-
	Other	-	-

### HE practices performed by parents

The results showed that all parents (100%; *n*=113) said that they felt comfortable to talk with nurses about issues related to the children/teenager’s health; 79.6% (*n*=90) of the parents considered that nurses were available to clarify all doubts related to the children/teenager’s health, and 20.4% (*n*=23) considered that they were not.

When questioned about HE practice planning, 61.9% (*n*=70) considered that nurses prepare the practice according to the needs, 23.9% (*n*=27) considered that it is done according to prepared scripts/standards, and 14.2% (*n*=16) considered that planning is based on improvisation.

**Fig. 1:**
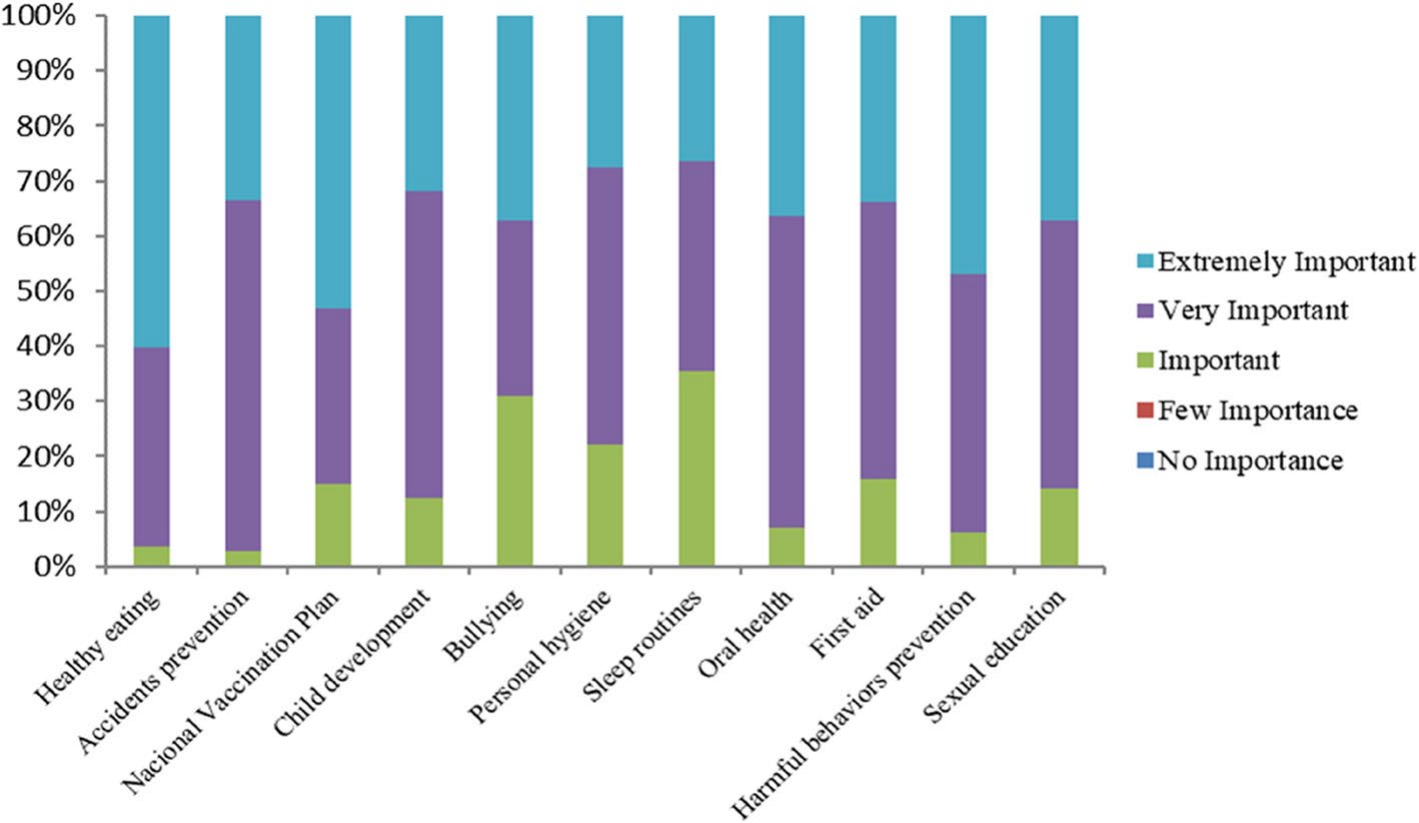
Most important issues discussed by nurses in HE practice. Northern Portugal, 2018, (*N*=133)

When questioned about the most important issue to be discussed during the HE practice with children/teenagers, the following themes were highlighted: "Healthy eating” (60.2%; *n*=68),"National Vaccination Plan" (53.1%; *n*=60) and "Harmful behaviors prevention" (46.9%; *n*=53) (Fig. 1).

When questioned about the importance of the HE practice, in comparison with the other nursing interventions, 66.4% (*n*=75) considered that it had the same importance as the other interventions; and 33.6% (*n*=38) considered that it had greater importance than the other interventions.

From the cross-referencing of HE practice importance delivered by nurses to children, teenagers and parents with “academic/professional qualifications”, no differences with statistical significance were found (χ²=8.915; df=4; *p*=0.063) (Table 3).

**Table 3:** Results of cross-referencing applied to the variable “academic/professional qualifications” and “HE practice importance” (chi-square test by Monte Carlo simulation). Northern Portugal, 2018, (*N* = 113)

	HE practice importance				Chi-square test		
Academic/professional qualifications		It has equal importance	It has greater importance	Total	Value	df	***Monte Carlo significance (2 sided)***
9th grade	Count	5ª	8^b^	13	8,915^a^	4	0,063
	Expected count	8,6	4,4	13,0			
	Adjusted residues	-2,3	2,3				
12th grade	Count	17ª	3ª	20			
	Expected count	13,3	6,7	20,0			
	Adjusted residues	1,9	-1,9				
Professional course	Count	8ª	6ª	14			
	Expected count	9,3	4,7	14,0			
	Adjusted residues	-0,8	0,8				
Graduation	Count	38ª	16ª	54			
	Expected count	35,8	18,2	54,0			
	Adjusted residues	0,9	-0,9				
Master	Count	7ª	5ª	12			
	Expected count	8,0	4,0	12,0			
	Adjusted residues	-0,6	0,6				
Total	Count	75	38	113			
	Expected count	75,0	38,0	113,0			
Likelihood Ratio					9,025	4	0,060
Fisher’s Exact Test					8,819		
Linear Association					0,381 ͨ	1	0,537

^a^3 cells (30,0%) have expected count less than 5. The minimum expected count is 0,27. ^b^Based on 10000 sample tables with initial value 624387341. ^c^The standardized statistics is -0.617

When analyzed the relationship between the HE practice importance given by nurses to children, teenagers and parents with “age”, it wasn’t found any statistically significant differences (χ²=6.816; df=3; *p*=0.061) (Table 4).

**Table 4:** Results of cross-referencing applied to the variable “age” and “HE practice importance” (chi-square test by Monte Carlo simulation). Northern Portugal, 2018, (*N* = 113)

	HE practice importance				Chi-square test		
Age		It has equal importance	It has greater importance	Total	Value	df	***Monte Carlo significance (2 sided)***
20-30	Count	14ª	6^a^	20	6,816^a^	3	0,061^b^
	Expected count	13,3	6,7	20,0			
	Adjusted residues	0,4	-0,4				
30-40	Count	42ª	14^a^	56			
	Expected count	37,2	18,8	56,0			
	Adjusted residues	1,9	-1,9				
40-50	Count	18ª	18^b^	36			
	Expected count	23,9	12,1	36,0			
	Adjusted residues	-2,5	2,5				
50-60	Count	1^a^	0^a^	1			
	Expected count	0,7	0,3	1,0			
	Adjusted residues	0,7	-0,7				
Total	Count	75	38	113			
	Expected count	75,0	38,0	113,0			
Likelihood Ratio					6,987	3	0,070^b^
Fisher’s Exact Test					6,580		0,064^b^
Linear Association					2,749^c^	1	0,125^b^

^a^2 cells (25,0%) have expected count less than 5. The minimum expected count is 0,34. ^b^Based on 10000 sample tables with initial value 957002199. ^c^The standardized statistics is 1,658

It is important to mention that although there’s no statistically significant relationship with academic/professional qualifications and with age, the analysis of the adjusted residuals, points out that in the group of [40-50[ years, there is a higher classification of HE as very important, which seems to indicate a greater appreciation of this practice by this age group.

The scale's HEAS analysis showed a percentage of parental agreement above 55% in all items, which can be revealing of a HE practice that is viewed in a positive and effective way, also capable of conscious, voluntary and health-promoting behaviors. In HEAS the highest average results was obtained in items which were related with a HE practice based in partnership of care. This result reflects the recognition of close relationship, involvement and participation of parents, their active role in HE practice and the ability to adopt responsible and conscious health behaviors, that is, parents feel the HE performed by nurses as a practice which enhances parental performance, increased health literacy and responsible decision-making by the children, teenagers and parents. The item with 100% of agreement by parents, were related with the adoption of healthy lifestyles, which highlights the importance attributed by nurses to health determinants with a view in preventives interventions.

The analysis of the association of the HEAS also revealed no statistically significant differences with "academic/professional qualifications" (*p*=0.126), nor with "age" (*p*=0.512).

## Discussion

The WHO recognizes the fundamental nurse’s role as well, but HP must start early, even because children should learn to make healthy choices. However, many programs lack in evaluation [17]. Thus, the assessment of parents (target of nurse’s care) is extremely important, as it may be an instrument used by nurses, to make their interventions more effective, as well as can be a good care quality indicator [17]. In this study, all respondents reported a trust climate with nurses which allows them to exchange impressions or doubts about their children's health status.

This result reveals that, although an effective intervention of the HE practice may have its own setbacks and challenges [3], nurses developed a practice based on health-promoting environments capable of creating mediation, negotiation and build a relationship of partnership and trust, making children, teenagers and parents feel that they are an essential part of the process, which inherently promotes a practice aimed at their health needs. Also, in a study about nurses and parent’s collaboration, it was found that it was based in dialogue, action, flexibility and reciprocity [18]. In a study about nurses' perception of educating parents about obesity, despite pointing out the lack of knowledge about what to offer to the parents, it was reported that dialogue is facilitated if it was built on trust [19].

This study also seems to point the effectiveness of the verbal and non-verbal communication, developed by nurses, and contribute to the awareness of the professional's performance health, and the efficiency and quality of health communication [20]. It should be noted that the communication process is one of the essential competencies in HP, defined by the pan-European project Developing Competencies and Professional Standards for Health Promotion Capacity Building in Europe (CompHP), of the European Office of the International Union for Health Promotion and Education [21], and, because of this, the nurses' communication patterns have also been studied in the literature [22].

In turn, communication techniques were pointed out by nurses as a formative need [23]. It was also verified that nurses pointed out the lack of confidence in their communication skills [19], and, in a study based on the way people engage with the news, which requires a review of communication guidelines (especially during public health crises that bring unique challenges). There were identified three areas of health communication empowerment: proactivity, planning ahead and centrality of the individual [24]. Therefore, we can state that it would be beneficial and important to deepen the theoretical understanding of this issue, whether in the initial training of nursing or in continuous training processes.

Assuming that the perception of individuals, in relation to HE practice developed by nurses, has a positive impact on the overall satisfaction of individuals [22], and the fact that, in the present study, most parents (79.6%; *n*=90) felt that nurses were time available to clarify their doubts, may be an indication of good care organization and time management, thus providing better access to health care, increased demand for nursing care and, consequently, health gains. Also, in a study on neonatal nursing, it was found that mothers showed a moderate level of satisfaction with the social support provided by nurses [25].

The results of this study can also reinforce the idea that nurses are the health professionals closest to the community, which allows them to identify problems/needs, promote the maintenance of children, teenagers, and parents’ health status, and thus, develop interventions which promotes an efficient HE practice. In this sense, the "*Programa Nacional de Saúde Infantil e Juvenil*" (National Program for Child and Youth Health), assumes that, in attending children health appointments, nurses develop an important role in health education and disease prevention with children, teenagers and parents [7].

Regarding the planning of HE interventions, most parents (61.9%; *n*=70) identified the nurses' flexibility in meeting their needs. This clear perception of parents about the nurses' assessment of their health needs leads to evidence of an HE practice based on adequate lines of action, directed and adapted to the binomial children, teenagers and parent’s complexity, in each child/teenager life cycle, and, consequently, capable of acting on health determinants [14]. It was also found, in a study, that the importance of knowing the individuals' problems contributed to the success of nurses' interventions [22].

Considering that behaviors and attitudes are important HE focuses, regarding the most important topic to be addressed in HE, 60.2% of parents (*n*=68) pointed out "Healthy eating". Other studies corroborate this result. When cross-referenced parent’s health literacy and children’s health behaviors, the children whose parents had high health literacy, ingested more salads, vegetables and fruit and practiced more physical exercise [5]. Regarding family influences on eating practices, it was demonstrated that these are preponderant in eating practices, hindering or facilitating healthy eating [26]. In a study about relationship between social support in social networks and risk factors for obesity, it was found that most adolescents did not eat properly, and the main cause indicated was the lack of motivation [27]. An international study with universities from several countries showed that the area where a large investment must be made was healthy eating [28].

Several studies show that the relationship between parents and health professionals is crucial for long-term weight maintenance in obese children [19]. Thus, the importance of promoting healthy lifestyles in partnership, performed by nurses, is evidenced, since it is not only important to promote the correct food choices (by the children/teenagers), but also empower parents, because childhood overweight is a public health problem, which has increased worldwide, and family influence is a conditioning factor [26]. In other words, there is a co-responsibility and conscious decision shaped by parents during the first 12 months of life, subsequently suffering the influence of external factors like friends, teachers and school environment [28].

It should be noted that, in this study, the "National Vaccination Plan" and the "Harmful behaviors prevention" were also considered important topics by 53.1% (*n*=60) and 46.9% (*n*=53) of parents, respectively. This result can also support the framework of the HE practice, performed by nurses with the children/teenagers and parents' health surveillance programs [7], with nursing interventions contributing to the empowerment of good practices and health decision-making by parents, since they are responsible for the children's health and well-being. Similarly, in other study, it was found a relationship between teenagers/children's health and parent’s health literacy [5].

In relation to the health professional to whom parents first resorted to clarify doubts, the "Pediatrician" was indicated by 56.6% (*n*=64) of parents. Also, in a study about the parents’ perspective of their children education in preoperative preparation, it was found that the doctor was seen as the main educator of the child and parents, rather than other health professionals, including nurses [29]. Assuming that, all health institutions and health professionals offer understandable and usable health information from the individuals’ perspective [23]. This result may be due to the preference for the health professional with more qualifications in child health field, and that is the pediatrician. Although nurses also point out the insufficient cooperation with other health professionals and organizational barriers, as factors that affect the relationship and educational support of parents [19], in a study about collaboration between nurses and parents, the experience of nurses was considered an important goal for quality of pediatric nursing care [30].

To allow a better assessment of the HE practice, the parents were questioned about the comparison between HE and other nursing interventions. Although HP is focused on individual behaviors based on a wide range of interventions, on social and environmental determinants, as well as other health-related aspects [31], most parents in the present study (66.4%; *n*=75), considered the HE practice equally important like the other interventions. There was also no relationship between sociodemographic variables (academic/professional qualifications: χ²=8.915; df=4; *p*=0.063; age: χ²=6.816; df=3; *p*=0.061) with the importance attributed to the HE practice, so it can be concluded that academic qualifications and age do not influence the parents’ assessment of HE practice provided by nurses to children, teenagers and parents. This result may suggest that, even though the age or academic qualifications the HE interventions are seen, by individuals, as a practice very focused on the transmission of health-promoting behaviors, the acquisition of healthy lifestyles is allowed [12].

Although several recent studies pointed parents’ health literacy as a part of health intervention [5], it’s necessary that health systems throughout the world respond to the changing needs of health population, by motivating nurses and other health professionals to maintain their professional development [32], highlight the importance of HE.

Since the evaluations of the implementation processes in HP provide an understanding of the usefulness strategies and guide decisions that maximize the success of community programs [33], the evaluation portrayed by the HEAS translated the of the HE practice contribution, performed by nurses, in a more comprehensive way. Therefore, it was verified that the HE practice was positively evaluated and seen as effective and enhancing conscious, voluntary and health-promoting behaviors.

It should be noted that the items with the highest percentage of agreement ("It allows the respect of the health decision-making adopted by parents" and "It allows the appreciation of parents as a structure with functions and resources that affects the children/teenager’s health and disease processes") reflect the recognition of the close relationship, the parents' involvement and participation, and their active role in the HE practice, which corroborates other studies. Good teamwork with parents is considered very important by nurses, in HP ethics care of school health nurses [34]. In a study of hospitalized children parent’s and their partnership with nurses, it was found that, the greater partnership, the higher quality of nursing care provided to the hospitalized child [35]. Also, in a systematic review about nurses and other health professionals’ perceptions in relation to parent education practice, it was pointed out that family-focused approaches allowed parents to practice and improve skills over time [36].

In this sense, based on the idea that to have health literacy, the understanding and correct use of health information is necessary, the promotion is essential to adjust the social environment in a pertinent way for the use of health services by individuals [23], which is denoted in the result of this study, since parents feel the HE performed by nurses as a practice based on a care partnership that’s improving parental performance, and increase health literacy and responsible decision-making.

It should be noted that, an item obtained full agreement and the highest mean of the HEAS ("Allows the healthy lifestyles adoption by children/teenagers/parents"), which reinforces the idea that nurses prioritize the dissemination of healthy lifestyles, which, consequently, generates health-promoting behaviors and makes HE practice as a support for behavioral change on children/teenagers and parents. In fact, this result is in line with the priority topics that should be part of HE ("Healthy eating"; "National Vaccination plan" and "Harmful behaviors prevention"), which also have a health promoting nature, revealing the importance attributed by parents in the adoption of behaviors that generate healthy lifestyles. Thus, the strengthening of person-centered health with the aim of accountability can provide the necessary support for the promotion of healthy adults [34]. However, in a study on parents' perceptions of their 6-year-old children's eating behaviors and physical activity, the parents considered that the children's healthy efforts and behaviors may be influenced by contextual circumstances and barriers, such as social norms and structures [37].

At least, the present study considered the opinion of those who is addressed the nursing care. So, it brought the recognition of a practice that values the partnership between nurses and children, teenagers and parents, that recognizes their needs, that allows the healthy lifestyles promotion, the respect for decision-making in health matters, and the adoption of health protective behaviors through strategies that motivate the maximum health potential of the children, teenagers and parents.

### Limitations of the study

The main limitation of this study results from the sample type (convenience), its geographical limitation and number of participants, that prevents the generalized results. Another limitation was the absence of already validated instruments, which forced us to build a new one, and although we tried to carry out validity and reliability studies (following the most current guidelines which pointed towards the validity of the instrument), we understand that it can be improved. On the other hand, taking into account the importance of the topic and the children's development, it would be interesting to apply the questionnaire (in the consultation room at the end of the health appointments), to teenagers, in order to provide the teenager’s assessment about HE practice carried out by nurses.

## Conclusion

This study shows that parents, once they feel comfortable to talk with nurses and consider they make the preparation according to the identified needs, they perceive the HE practice as adaptable to the complexity of the children, teenagers and parents’ binomial. By identifying important subjects to be approached (healthy eating, National Vaccination Plan and harmful behaviors prevention), parents' collaboration is important for nurses because it facilitates the intervention on health determinants and the promotion of practices that protect, maintain and/or improve children, teenagers and parents’ health. Furthermore, this parents' assessment recognized the nurses' availability to provide proactive responses according to the needs, thus translating into health gains and the recognition of this practice. The results may serve as a reference because, in addition to assessing the impact of the HE strategies, they may be useful to reflect critically and strategically on this area and be able to (re)formulate and/or improve the HE interventions to children, teenagers and parents, even because the parents considered this practice as equal important compared with other nursing interventions.

Finally, we believe that this study produced results, which highlights the nurses' position as health educators, and the importance of other researches to continuing to give visibility to this practice, and its contribution in maintaining the continuous improvement of nurses’ practice quality.

## Data Availability

The authors did not want share raw data of the study, because it was obtained an informed consent from all subjects involved in the study, and it was guaranteed the anonymity and confidentiality.
